# Prevalence of intestinal parasitic infection and associated risk factors among village health volunteers in rural communities of southern Thailand

**DOI:** 10.1186/s12889-017-4486-2

**Published:** 2017-06-09

**Authors:** Chuchard Punsawad, Nonthapan Phasuk, Suchirat Bunratsami, Kanjana Thongtup, Niramon Siripakonuaong, Somchok Nongnaul

**Affiliations:** 1 School of Medicine, Walailak University, 222 Thasala District, Nakhon Si Thammarat, 80161 Thailand; 2 Tropical Diseases and Parasitic Infectious Diseases Research Group, Walailak University, 222 Thasala District, Nakhon Si Thammarat, 80161 Thailand; 3 Noppitum Hospital, Noppitum District, Nakhon Si Thammarat, 80161 Thailand

**Keywords:** Intestinal parasitic infection, Village health volunteers, Risk factors, Nakhon Si Thammarat Province, Thailand

## Abstract

**Background:**

Intestinal parasitic infections remain prevalent and constitute a public health problem in certain rural areas of Thailand. Village health volunteers (VHVs), who are members of a Thai healthcare alliance, function as key providers of health prevention measures, disease control, and health education and share national health promotion campaigns with community members. This study is aimed at evaluating the prevalence, intensity, and risk factors for intestinal parasitic infection in VHVs in order to design community awareness and health education campaigns for the target population.

**Methods:**

This cross-sectional study was conducted between January to April 2016 among village health volunteers (VHVs) from four sub-districts of Nopphitam District, Nakhon Si Thammarat Province, southern Thailand. Subjects for the study were selected using a simple random sampling method. Socio-demographic variables and risk factors were collected by a structured questionnaire. Stool specimens were collected and processed using direct wet mount and formol-ether concentration techniques to determine the presence of parasites and modified Kato-Katz thick smear to determine the intensity of infection.

**Results:**

A total of 324 VHVs were enrolled. The overall prevalence of intestinal helminths was 9.3% (95% confidence interval [CI]: 6.3–13.0). The prevalence of hookworm, *Strongyloides stercoralis*, and *Trichuris trichiura* were 8.0% (95% CI: 5.3–11.5), 0.9% (95% CI: 0.2–2.7), and 0.3% (95% CI: 0–1.7), respectively. Mean intensity of hookworm infection was 1732 eggs per gram of stool. The prevalence was lower for protozoan infection than for helminth infection. *Blastocystis hominis* accounted for the highest percentage of intestinal protozoan infections 4.0% (95% CI: 2.2–6.8), followed by *Giardia intestinalis* 0.6% (95% CI: 0–2.2). No statistically significant difference was observed in the prevalence of intestinal parasitic infection among sub-districts (*p* > 0.05). Having dogs at home was associated with soil-transmitted helminth (STH) infection in study participants (Crude prevalence ratio [CPR]: 2.3; 95% CI: 1.0–5.2).

**Conclusions:**

This study is the first to describe the prevalence of intestinal parasitic infection VHVs from southern Thailand. Hookworm infection is more prevalent than other types of STH infection. The development of community awareness campaigns and appropriate control measures should be considered to reduce the prevalence of hookworm infection, especially among VHVs who are the key persons providing health education to the community.

**Electronic supplementary material:**

The online version of this article (doi:10.1186/s12889-017-4486-2) contains supplementary material, which is available to authorized users.

## Background

The World Health Organization estimates that approximately 1.5 billion people are infected with soil-transmitted helminths worldwide [1]. In Thailand, helminth infection remains a significant health problem in rural communities of some regions. A national survey in 2009 found the prevalence of helminthiasis in the Thai population was 18.1%, with persistent high prevalence of opisthorchiasis and hookworm infection in the northeastern and southern regions of Thailand, respectively [2]. The high prevalence of intestinal parasitic infection is closely associated with poverty, climatic conditions, poor personal hygiene, poor sanitation, and unsafe drinking water [2–5].

Previous studies conducted in areas of southern Thailand have given due attention to distributions of intestinal parasites in adults and school-age children [2, 6, 7], but patterns of intestinal parasitism have not been reported in village health volunteers (VHVs) who act as community health workers in rural communities of Thailand. VHVs are members of a Thai healthcare alliance established to promote healthcare service communication and collaboration at the primary healthcare level. In general, VHVs are local folks who willingly volunteer themselves to work for their community in terms of public health. Apart from their occupations, they generally act as a mediator between local healthcare providers and local folks. All VHVs receive health information from public health personnel and function as key providers of information about health promotion and education, disease control, and available basic health services to villagers [8]. To understand STH burden in VHVs would be helpful not only in determining the burden of the infection in the community as they are local folks themselves, but also in providing awareness of STH and the importance of personal hygiene as they are groups of people who aid the local healthcare personnel providing health education to the community.

Baseline data on intestinal parasitic infection is necessary to plan appropriate control programs and strategies for VHVs. Therefore, the purpose of this study is to determine the prevalence and associated risk factors of intestinal parasitic infection in VHVs living in Nopphitam District, Nakhon Si Thammarat Province, southern Thailand. The findings of this study help strengthen the information currently available about parasitic infections and can be used to encourage policy makers and public health officials to develop training programs for parasitic control and health promotion for community health workers.

## Methods

### Study design and setting

**Fig. 1:**
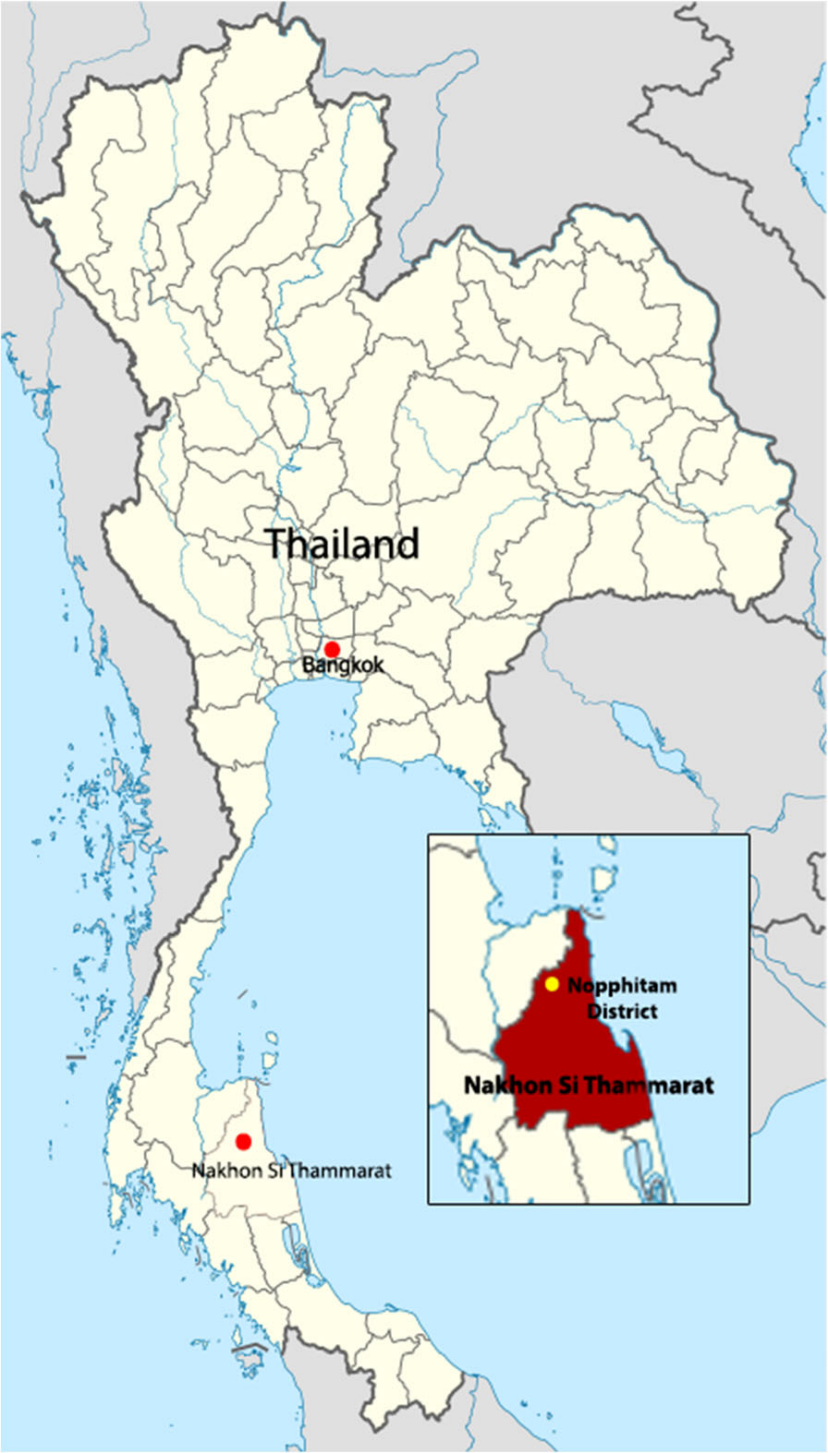
Map of Nopphitam District, Nakhon Si Thammarat Province, southern Thailand. (Map from Wikimedia Commons: https://commons.m.wikimedia.org/wiki/Atlas_of_Thailand#)

This cross-sectional study was carried out from January to April 2016 in Nopphitam District, Nakhon Si Thammarat Province, southern Thailand. Nopphitam District is located at the geographical coordinates of 8°43′10″N latitude and 99°45′6″E longitude, which is about 780 km south of the Thai capital of Bangkok (Fig. 1). Average temperature is 27.1 °C, with a minimum 25.8 °C in January and maximum 29.3 °C in May. Annual rainfall is 1454.3 mm (Climatological Center, Thai Meteorological Department, Annual Report 2015). This district is divided into four sub-districts (tambon), namely Na Reng, Karo, Nopphitam, and Krungching, which are further subdivided into 38 villages (muban). Nopphitam District is considered remote and rural and there is only one community hospital. These four sub-districts share the same geographic landscape. The main access road is non-asphalt while the remaining roads are asphalt. The majority of land is used for farmland and rubber plantations. According to the Department of Provincial Administration, the estimated total population of these sub-districts in 2015 was 33,183. Agriculture is the main economic activity of people in this area.

### Study population, sample size and sampling technique

The study was conducted among village health volunteers residing in four sub-districts of Nopphitam District, Nakhon Si Thammarat Province. The sample size was determined using the single population proportion formula. It was calculated from the prevalence rate (p) of 19.8% from a previous study [2], with a 95% confidence interval (z = 1.96), 5% margin of error (d = 0.05). The calculated sample size was 244. We assumed that the final sample size would be reduced by around 30% due to unable to pass stool on the study date and thus aimed for a sample size of 330. Simple random sampling method was used to selected village health volunteers from each sub-districts. We randomly selected 330 VHVs, then gave instructions and distributed plastic containers for stool collection. Finally, a total of 324 village health volunteers returned stool specimens, which consisted of 22.22% (72) from Na Reng, 24.38% (79) from Karo, 16.98% (55) from Nopphitam, and 36.42% (118) from Krungching. We decided to keep all the samples in the study for the sake of health surveillance regardless of the calculated sample size, hence, the final sample size was 324.

### Data collection and laboratory processing

A structured questionnaire was developed and used to collect demographic and socioeconomic data (i.e. age, education level and income). Information on possible risk factors (i.e. personal hygiene behaviors such as hand washing and shoe wearing) was also collected via the questionnaire (Additional file 1). All completed questionnaires were checked for accuracy and completeness. After proper instruction, a clean plastic container labeled with the subject’s name and identification number was distributed to each participant and one day later they returned containers back with at least 10 g of stool. Each specimen was checked for correct labeling, quantity of sample, and procedure of collection and transported immediately to the parasitology laboratory of the School of Medicine, Walailak University.

A portion of each specimen was processed immediately using a direct microscopic technique with a saline and iodine solution to detect cysts, trophozoites, eggs, and larvae of intestinal parasites. The remainder of each specimen was analyzed using the formol-ether concentration technique. Briefly, 1 g of stool sample was suspended in 3 ml of 10% formalin, sieved with cotton gauze, and transferred to a 15-ml conical centrifuge tube. Then, 8 ml of 10% formalin and 3 ml of diethyl ether were added and the resulting solution was centrifuged for 5 min at 2000 rpm. Subsequently, the supernatant was discarded, and the remaining sediment was observed under light microscope at 10× and 40× objectives to detect eggs and larvae of helminths, cysts, and trophozoites of protozoan parasites. Iodine solution was used to detect and identify cysts of protozoan parasites. In addition, each stool sample was analyzed by the modified Kato-Katz technique [9] (kits were purchased from Department of Helminthology, Faculty of Tropical Medicine, Mahidol University). The intensity of soil-transmitted helminth (STH) infection was estimated indirectly by counting the number of eggs per gram (EPG) of stool and calculated by multiplying each slide count by 40. The results of intensity for STH were classified into low, moderate, and high according WHO recommendation; for *Ascaris lumbricoides*: low 1–4999 EPG, moderate 5000–49,999 EPG, and high 49,999 EPG; for *Trichuris trichiura*: low 1–999 EPG, moderate 1000–9999 EPG, and high 9999 EPG; and for hookworm; low 1–1999 EPG, moderate 2000–3999 EPG, and high 3999 EPG, respectively [10]. To eliminate observer bias, each stool sample was examined by two trained senior medical laboratory technologists who were not informed about the health status and other details of the study participants.

### Data analysis

We used proportions with 95% confidence intervals (CIs) to describe the prevalence of intestinal parasites. Quantitative variables were described by mean and standard deviations (SD) and qualitative variables were described by frequency (percentage). Initially, the differences of the presence of intestinal parasites among sub-districts and were assessed by chi-square test. The prevalence ratios were computed by Poisson regression with robust estimation to measure the strength of the association between each independent variable and STH infection. To adjust for possible confounders, all variables with *P*-value less than 0.1 in the univariate analysis were analyzed using multivariate Poisson regression analysis. *P*-value less than 0.05 was considered statistically significant. Data were entered, cleaned, and analyzed using IBM SPSS Statistics for Windows, Version 23.0. Armonk, NY: IBM Corp.

## Results

### Socio-demographic characteristics

A total of 324 study participants, comprising 38 males and 286 females, were enrolled in the study. The mean age of participants was 45 years (standard deviation 9.0), with a range of 23 to 71 years. The age-group distribution showed that a majority (39.5%) were in the 41–50-years age group. All participants were Buddhist. Most participants were married (89.5%), and a majority was farmers (80.2%). About half (53.1%) of participants indicated secondary education was the highest level received, and around one-third (32.4%) indicated primary education was the highest level (Table 1).

**Table 1:** Socio-demographic characteristics of study participants in Nopphitam District of Nakhon Si Thammarat Province, Thailand

Characteristics	Number	Percentage
Sex		
Male	38	11.7
Female	286	88.3
Age group		
21–30	19	5.9
31–40	85	26.2
41–50	128	39.5
≥ 51	92	28.4
Marital status		
Single	8	2.5
Married	290	89.5
Divorced	21	6.5
Widowed	5	1.5
Education		
Primary school	105	32.4
Secondary school	172	53.1
High vocational/college certificate	26	8.0
Bachelor’s degree or over	21	6.5
Occupation		
Farmer	260	80.2
Employee	21	6.5
Merchant	14	4.3
Housewife	20	6.2
Other	9	2.8

### Prevalence of intestinal parasitic infection in village health volunteers

The overall prevalence of intestinal parasitic infection among participants was 45/324 (13.9%, 95% CI: 10.3–18.1). A majority of the infected participants 97.8% (44/45) had a single infection while one participant 2.2% (1/45) was found to have a double infection (hookworm and *Trichuris trichiura*). Among helminth infections, hookworms 27/324 (8.3%, 95% CI: 5.6–11.9) was the predominate parasite, followed by *Strongyloides stercoralis* 3/324 (0.9%, 95% CI: 0.2–2.7) and *T. trichiura* 1/324 (0.3%, 95% CI: 0–1.7). No cestodes or trematodes were detected in this study. Of 27 participants who were positive for hookworm infection, 85.2% (23/27), 11.5% (3/27), and 3.7% (1/27) had low (1–1999 EPG), moderate (2000–3999 EPG), and high (≥4000 EPG) intensity of infection, respectively. All participants infected with *T. trichiura* had low (1–999 EPG) intensity.

The prevalence of helminth infection ranged from 7.6% to 11.4% at the study sites. The highest prevalence of the helminth infection was observed in Karo sub-district, and protozoan infection was found frequently in Krungching sub-district. The overall difference in parasite prevalence among the study sites was not statistically significant (*p* > 0.05) (Table 2). The prevalence of helminth infection was twice the prevalence of protozoan infection. With regard to protozoa infection, the prevalence of protozoan infection ranged from 1.8% to 5.9%. The highest prevalence was due to *Blastocystis hominis* 13/324 (4.0%, 95% CI: 2.2–6.8) and followed by *Giardia intestinalis* 2/324 (0.6%, 95% CI: 0–2.2) (Table 2).

**Table 2:** Prevalence of intestinal parasitic infection in Nopphitam District, Nakhon Si Thammarat Province, Thailand

Parasite	Study site				Overall (*n* = 324)	*X* ^*2*^	*P*-value
	Na Reng	Karo	Nopphitam	Krungching			
	(*n* = 72)	(*n* = 79)	(*n* = 55)	(*n* = 118)	No. (%)		
	No. (%)	No. (%)	No. (%)	No. (%)			
Helminths							
Hookworms	7 (9.7)	7 (8.8)	3 (5.4)	9 (7.6)	26 (8.0)	0.798	0.850
*Strongyloides stercoralis*	0 (0.0)	1 (1.3)	2 (3.6)	0 (0.0)	3 (0.9)	5.472	0.140
*Trichuris trichiura*	0 (0.0)	1 (1.3)	0 (0.0)	0 (0.0)	1 (0.6)	2.418	0.491
Total	7 (9.7)	9 (11.4)	5 (9.0)	9 (7.6)	30 (9.6)	0.048	0.997
Protozoans							
*Blastocystis hominis*	2 (2.8)	4 (5.1)	1 (1.8)	6 (5.1)	13 (4.0)	2.283	0.516
*Giardia intestinalis*	1 (1.4)	0 (0.0)	0 (0.0)	1 (0.8)	2 (0.6)	2.349	0.503
Total	3 (4.2)	4 (5.1)	1 (1.8)	7 (5.9)	15 (4.6)	1.648	0.648
Overall	11 (15.3)	13 (16.4)	6 (10.9)	16 (13.5)	45 (13.8)	0.698	0.874

**Fig. 2:**
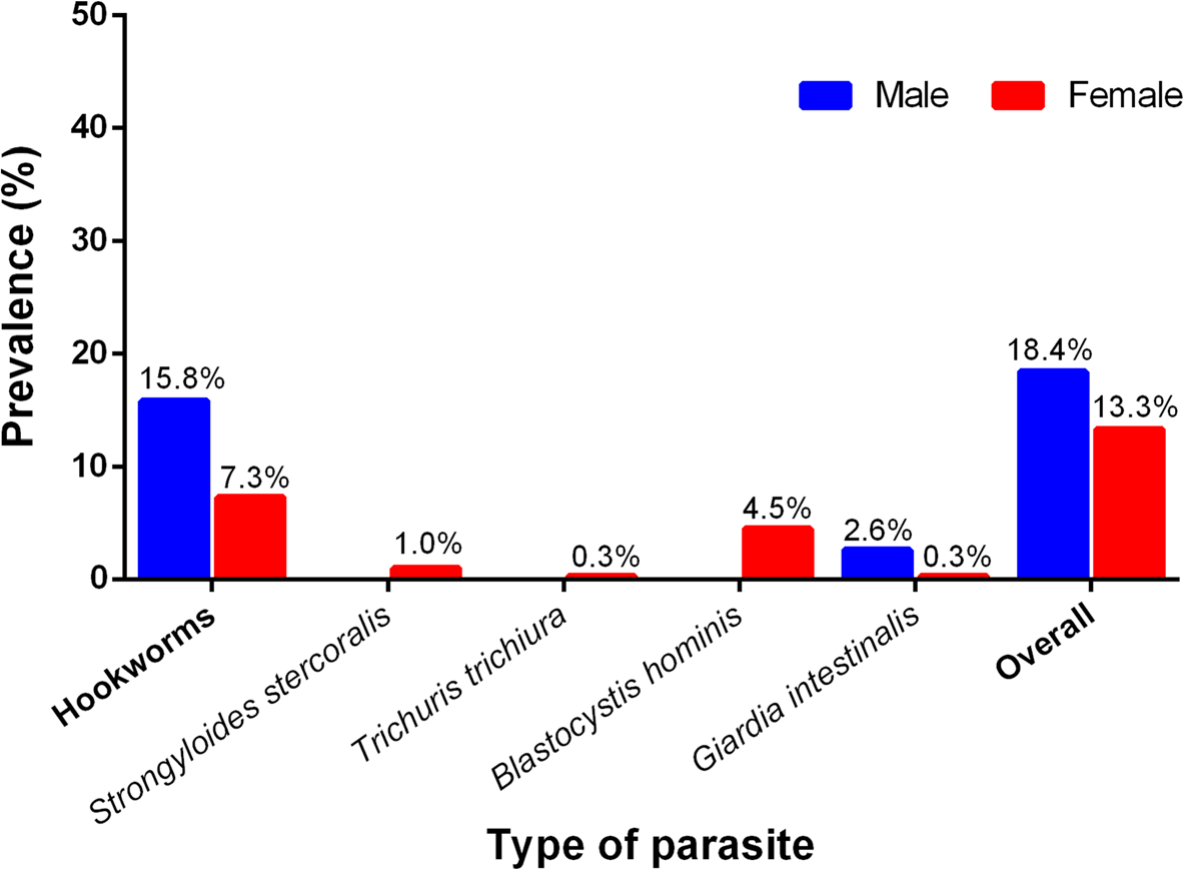
Sex-related prevalence of intestinal parasites among study subjects in Nopphitam District, Nakhon Si Thammarat Province, Thailand

The distribution of infection among female and male participants is shown in Fig. 2. Male participants had the overall prevalence at 18.4% (7/38) whereas the infection rate in female participants was slightly lower at 13.3% (38/286). The difference in prevalence of intestinal parasites between female and male participants was not statistically significant.

### Risk factors associated with soil-transmitted helminth (STH) infection

Univariate analysis of factors potentially associated with STH, as shown in Table 3, revealed significant associations between STH infections with having dogs at home. VHVs who had dogs at home were two times more likely to be infected than those who did not (Crude prevalence ratio 2.3; 95% CI: 1.0–5.2, *p* = 0.047). STH infection was not significantly associated with gender, age group, or monthly family income (*p* > 0.05). All the participants with STH infection graduated the highest level of education at primary/secondary school. The prevalence ratio regarding level of education could not be calculated due to none of participants with bachelor’s degree or higher had STH infection. After adjusting for possible confounders by multivariate analysis, none of the risk factors were associated with STH infection (Table 3).

**Table 3:** Univariate and multivariate analysis of soil-transmitted helminth infection and potential risk factors among study participants

Variables	Positive No. (%)	Negative No. (%)	Total *n* = 324	Crude PR (95% CI)	*P*-value	Adjusted PR (95% CI)	*P-*value
Gender							
Male	6 (15.8)	32 (84.2)	38	1.9 (0.8–4.3)	0.135		
Female	24 (8.4)	262 (91.6)	286				
Age in years							
< 40	6 (6.6)	85 (93.4)	91	0.6 (0.3–1.5)	0.310		
≥ 40	24 (10.3)	209 (89.7)	233				
Monthly family income (Thai Baht)							
< 10,000	14 (11.0)	113 (89.0)	127	1.4 (0.7–2.7)	0.380		
≥ 10,001	16 (8.1)	181 (91.9)	197				
Education							
Primary/secondary school	30 (9.9)	273 (90.1)	303				
Bachelor’s degree or higher	0 (0)	21 (100)	21				
Wears shoes when outside							
No	1 (20.0)	4 (80)	5	2.2 (0.4–13.1)	0.387		
Yes	29 (9.1)	290 (90.9)	319				
Wear boots when on farm							
No	1 (14.3)	6 (85.7)	7	1.6 (0.3–9.9)	0.636		
Yes	29 (9.1)	288 (90.9)	317				
Open-field defecation							
Yes	29 (11.0)	235 (89.0)	264	6.6 (0.9–47.4)	0.061	5.5 (0.7–41.2)	0.099
No	1 (1.7)	59 (98.3)	60				
Have dogs at home							
Yes	23 (12.0)	168 (88.0)	191	2.3 (1.0–5.2)	0.047	1.6 (0.7–3.7)	0.276
No	7 (5.3)	126 (94.7)	133				
Have cats at home							
Yes	12 (8.7)	126 (91.3)	138	0.9 (0.5–1.8)	0.763		
No	18 (9.7)	168 (90.3)	186				
Contact with domestic animals							
Yes	27 (11.2)	215 (88.8)	242	3.1 (1.0–9.8)	0.061	2.5 (0.8–7.8)	0.131
No	3 (3.7)	79 (96.3)	82				
Wash hands after touching animals							
No	1 (10.0)	9 (90.0)	10	1.1 (0.2–7.2)	0.934		
Yes	29 (9.2)	285 (90.8)	314				
Drinks purified water							
Not boiled or filtered	10 (14.3)	60 (85.7)	70	1.8 (0.9–3.7)	0.101		
Boiled or filtered	20 (7.9)	234 (92.1)	254				

## Discussion

This study demonstrates the prevalence of intestinal parasite infection among VHVs living in Nopphitam District of Nakhon Si Thammarat Province, southern Thailand. The overall prevalence of intestinal parasitic infection 45/324 (13.9% 95% CI: 10.3–18.1) was lower than the prevalence found in other similar studies. For example, a survey of residents of southern Thailand found prevalence of 19.8% [2], and a survey conducted in rural communities in northeast Thailand found 37% of people had an intestinal parasitic infection [3]. These variations in prevalence may be due to differences in climatic conditions, environmental sanitation, economic and educational status of study subjects, and previous control efforts. In the present study, multiple infections were found in only one subject (2.2% of the sample), which is much lower than the prevalence determined in previous studies in rural Thai communities [3, 5]. Differences in the study population, sample size, and methods of analysis could contribute to differences in the detection of various parasites. The present study was conducted among VHVs, which may have resulted in participants with better knowledge of personal health and hygiene than other study populations, thus contributing to lower prevalence of intestinal parasitic infection. As we enrolled 324 VHVs which exceeded the calculated sample size, our findings were considered to represent the population in this area in terms of the generalizability.

For protozoan infection, the prevalence of *Blastocystis hominis* and *Giardia intestinalis* infection in this study was 4.0% and 0.6%, respectively. Infection with *Blastocystis hominis* was more prevalent among female participants. Among sub-districts, Krungching sub-district had slightly higher prevalence of protozoa infection when compared with others. In general, all sub-districts are considered homogeneous in terms of geography and general living conditions. Although the highest prevalence of protozoa was observed at Krungching sub-district, there was no statistical difference. These pathogenic protozoa are transmitted by the fecal-oral route. *Blastocystis hominis* can cause diarrhea if the burden of infection is high. The presence of five or more *B. hominis* cells per 40× magnification field during direct microscopic examination of a wet-mounted stool smear is considered a sufficient amount of this pathogen to cause clinical illness [11]. *Blastocystis* sp. is transmitted through consumption of untreated or minimally treated water and uncooked or undercooked food [12]. Therefore, the burden of pathogenic intestinal protozoa causing diarrhea in the communities comprising the study area should be further studied, and water sources and supplies in the community should be further examined for pathogenic intestinal protozoa. Molecular techniques such as polymerase chain reaction are needed to confirm prevalence and identify subtypes of *Blastocystis* sp.

STHs are a group of parasitic nematode worms causing human infection through contact with eggs or larvae that thrive in the warm and moist soil of tropical and subtropical areas [13]. Globally, an estimated 438.9 million people were infected with hookworm, 819.0 million with *Ascaris lumbricoides*, and 464.6 million with *T. trichiura* in 2010 [14]. STHs, including hookworms, *S. stercoralis*, and *T. trichiura*, were detected in study participants whereas *A. lumbricoides* was not. This finding is consistent with a previous study among highland and lowland dwellers in the Gamo area of southern Ethiopia [15]. On the contrary, other studies conducted in peninsular Malaysia reported *T. trichiura* as the predominant STH in children [16, 17]. High prevalence of *S. stercoralis* was found in suburban areas of Nakhon Ratchaima Province in northeastern Thailand [5] and in rural areas of Cambodia [18, 19]. However, previous studies found *A. lumbricoides* was the most prevalent STH in school children in Ethiopia [20, 21], suggesting that young age might be associated with a higher risk of ascariasis. In addition, this study revealed that contact with domestic animals increased the risk of STH infection. However, data analysis revealed that the incidence of the STH infection was not significantly different among occupations. The differences in prevalence and distribution of STH among the various communities may be due to variations in host, age, genetics, time of study, parasitological technique used, personal hygiene practices, and environmental factors such as climate, topography, surface temperature, altitude, soil type, and rainfall, all of which significantly influence the distribution of STH [13, 22, 23].

STH is known to be endemic in southern Thailand. The present study showed that hookworm infection (8.3%) was more prevalent than other types of STH infection. However, the prevalence of hookworm infection was lower than in previous studies conducted in southern Thailand [2, 7, 24]. A recent national survey found the prevalence of hookworm infection was 15.8% in southern Thailand compared to 4.7%, 3.8%, and 3.2% in northeastern, north, and central Thailand, respectively [2]. The lower prevalence of hookworm infection in the present study may be due to differences in study population, urbanization, climate, general living conditions, and the accessibility of health services. In general, the majority of people infected with hookworms, *Strongyloides stercoralis*, and *Trichuris trichiura* are without symptoms. The participants who found to be infected with these parasites were informed and treated following CDC recommendations regarding the types of parasites. In Thailand, the costs of the anti-parasitic drugs are generally inexpensive.

This study demonstrated significant associations between STH infection and having dogs at home. Humans acquire hookworms when the infective larval stage (known as third-stage larvae or L3) living in soil either penetrates the skin or is ingested [25, 26]. In general, contact with dogs is not considered a risk factor for STH infection as they are not in the life cycle of the parasites. Interestingly, the association we found could result from soil contamination from dogs to humans. Furthermore, all participants with STH infection had the highest level of education of primary/secondary school whereas none of those graduated Bachelor’s degree or higher had STH infection. This implied that health education was needed to be emphasized among VHVs with lower degree of education. In Thailand, *Necator americanus* is the most common type of hookworm in the central [4], northeastern, and southern regions [7, 27]. In addition, *Ancylostoma ceylanicum* and *Ancylostoma caninum*, which are types of hookworm found in dogs and cats, have been detected in rural communities in Thailand [4, 27]. Therefore, the high percentage of study participants who indicated they keep pets at home and have contact with domestic animals indicates they may be at risk for acquiring animal hookworms. However, further work is needed to identify hookworms in the feces of domestic animals and in soil samples in communities in the studied area. These results may be helpful for understanding the mode of hookworm transmission and developing appropriate measures for controlling human and animal hookworm infection.

The present study was subjected to the following limitations. Fecal examination to detect the presence of helminths was carried out on only a single day, which could result in random error from one-time measurement and could affect diagnostic sensitivity for the detection of any parasites. Methods such as Harada Moori’s filter paper for *S. stercoralis* and hookworm infection were not performed. The risk factor analysis was restricted to questionnaire data and may not represent overall risk patterns. Unmeasured confounding in this study was presumably resulted from a selection bias. As the study was conducted among VHVs, it might probably select the participants with better knowledge of personal health and hygiene than other villagers. Finally, there were some limitations in the generalizability of our findings: due to the majority of VHVs’ gender, the gender of the participants was mainly female. Although gender was not statistically associated with STH infection, but the result of this study might not exactly represent the general population. Furthermore, the results of this study were applied to Nopphitam District, Nakhon Si Thammarat Province, Thailand, so there might be different results in other geographical settings, especially those outside southern Thailand.

## Conclusions

This is the first report to describe the prevalence of parasitic infections in village health volunteers from southern Thailand. Hookworm infection was prevalent in the studied communities. Having dogs at home was found to be significantly associated with STH infection. Appropriate measures such as education on personal hygiene and environmental sanitation and the development of awareness strategies should be implemented to control parasitic infection in VHVs.

## Additional file

Additional file 1: Questionnaire on demographic data and possible risk factors. (PDF 344 kb)

## Abbreviations

APR: Adjusted prevalence ratio
CI: Confidence interval
CPR: Crude prevalence ratio
EPG: Eggs per gram
STH: Soil-transmitted helminth
VHV: Village health volunteer
