# Supplementary material for: Prevalence of intestinal parasitic infection and associated risk factors among village health volunteers in rural communities of southern Thailand
Source: BMC Public Health. 2017 Jun 9;17:564. doi: 10.1186/s12889-017-4486-2 (PMC5466724; doi:10.1186/s12889-017-4486-2)
Supplement: Additional file 1: — Questionnaire on demographic data and possible risk factors. (PDF 344 kb) [file 12889_2017_4486_MOESM1_ESM.pdf]

Additional file 1: Questionnaire

Part 1: Demographic and socioeconomic data

1. Code no…………………

2. Sex ❍ 1. Male ❍ 2. Female

3. Age …………. Years

4. Sub-district
   - ❍ 1. Na Reng ❍ 2. Nobphitam
   - ❍ 3. Karo ❍ 4. Krungching

5. Religion
   - ❍ 1. Buddhism ❍ 2. Christian
   - ❍ 3. Islam ❍ 4. Other………………

6. Marital status
   - ❍ 1. Single ❍ 2. Married
   - ❍ 3. Widowed ❍ 4. Divorced

7. What is the highest level of education you have completed?
   - ❍ 1. Primary school
   - ❍ 2. Secondary school
   - ❍ 3. High vocational/college certificate
   - ❍ 4. Bachelor's degree
   - ❍ 5. Higher than bachelor's degree

8. Occupation
   - ❍ 1. Farmer ❍ 2. Employee
   - ❍ 3. Government officer ❍ 4. Merchant
   - ❍ 5. Housewife ❍ 6. Unemployed
   - ❍ 7. Other………………

.

9. Family income (per month)
   - ❍ Less than 10,000 Baht ❍ 10,001-20,000 Baht
   - ❍ 20,001-30,000 Baht ❍ 30,001-40,000 Baht
   - ❍ Over 40,000 Baht

10. Do you have pets at home? Please specify
    - ❍ 1. Dog(s)
    - ❍ 2. Cat(s)
    - ❍ 3. Other…………
    - ❍ 4. I don't have any pets at home.

Part 2: Information on possible risk factors

1) Do you wear shoes when going outside the house?

❍ 1. Never/seldom ❍ 2. Regularly

2) Do you wear boots for farm works?

❍ 1. Never/seldom ❍ 2. Regularly

3) You always drink water which is…………............

❍ 1. Not boiled or filtered ❍ 2. Boiled

❍ 3. Filtered ❍ 4. Purified by………………………

4) In a week, do you contact with domestic animals?

❍ 1. Never/seldom ❍ 2. Regularly

5) Do you wash hands after touching animals?

❍ 1. Never/seldom ❍ 2. Regularly

6) Do you practice open-field defecation?

❍ 1. Never/seldom ❍ 2. Regularly

7) Do you wash hands before meal?

❍ 1. Never/seldom ❍ 2. Regularly

8) Do you wash hands after defecation?

❍ 1. Never/seldom ❍ 2. Regularly

☺Thank you for your participation☺
